# Rare Mutation in Cystic Fibrosis as a Cause of Early-Onset Liver Disease and Esophageal Varices

**DOI:** 10.7759/cureus.78408

**Published:** 2025-02-03

**Authors:** Haneen M Toma, Amal R Al-Naimi, Khaled Abouhazima

**Affiliations:** 1 Pediatric Pulmonology, Sidra Medicine, Doha, QAT; 2 Pediatric Gastroenterology, Sidra Medicine, Doha, QAT

**Keywords:** 1677delta gene, children cystic fibrosis, cystic fibrosis, cystic fibrosis liver disease, pediatric

## Abstract

Cystic fibrosis liver disease (CFLD) is a common complication of cystic fibrosis (CF), typically emerging within the first two decades of life. It significantly impacts both short- and long-term prognosis, being the third leading cause of mortality in this population. We present the case of a child with a rare CF mutation who was diagnosed with CFLD, portal hypertension, esophageal varices, and early-onset CF-related diabetes at the age of 6. This case provides valuable insights into the early onset and progression of CF liver disease, highlighting the importance of timely diagnosis and management.

## Introduction

Cystic fibrosis (CF) is an autosomal recessive disorder characterized by impaired function of the CF transmembrane conductance regulator (CFTR) protein, which serves as a chloride channel in epithelial cells of the lungs, sweat glands, liver, pancreas, and intestines. Common symptoms of CF include meconium ileus, recurrent lung infections, bronchiectasis, pancreatic insufficiency, biliary cirrhosis, and poor weight gain, among others.

Liver disease, known as cystic fibrosis liver disease (CFLD), is a well-recognized manifestation of CF, typically developing within the first 20 years of life. In many cases, CFLD follows a stable or mildly progressive course later in life. Although most children with CF experience some degree of steatosis, clinically significant liver disease occurs in fewer than 10% of pediatric CF patients, usually by age 10. CF-related cirrhosis predominantly affects children and adolescents. When cirrhosis and/or portal hypertension are present, the condition is classified as severe CFLD [1]. The diagnosis of CFLD carries significant implications for both the short- and long-term prognosis of CF patients, making it the third leading cause of mortality in this population.

Here, we present the case of a child with a rare CF mutation (homozygous for the 1677delTA gene mutation), diagnosed at age 6 with CFLD, portal hypertension, and esophageal varices. While information regarding this genetic mutation is limited, it has been associated with increased rates of growth retardation, liver damage, and CF-related diabetes (CFRD), as well as a lower prevalence of chronic respiratory issues.

## Case presentation

A six-year-old Palestinian girl with CF was referred to our center for review. She had been diagnosed at three months of age with CF based on high sweat chloride levels and positive genetic testing for a pathogenic variant in the CFTR 1677delTA gene. The child had a history of intermittent wet cough, chronic constipation, and persistent abdominal distension since the age of 3. Prior to transfer, she was not on regular medication due to a lack of medical supplies.

On admission, she presented with stunted growth, a BMI of 15 kg/m² (z-score: -1.4). She showed no signs of pallor, jaundice, or clubbing. Lung examination revealed equal air entry bilaterally, with crackles in the right lower zone. Abdominal examination was notable for generalized distension, dilated abdominal wall veins, positive shifting dullness, and splenomegaly, with no lower limb edema.

**Figure 1:**
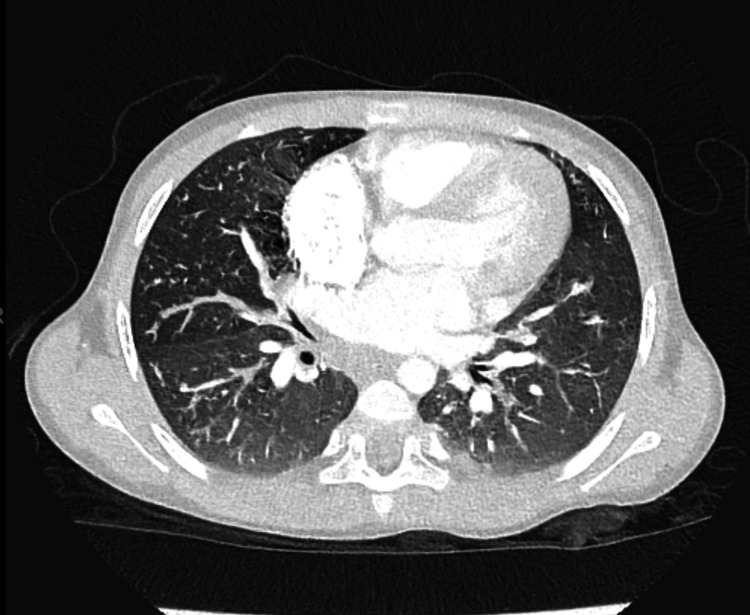
Chest CT showing mucus impaction

During her admission, she was diagnosed with incomplete distal intestinal obstruction, based on clinical and radiological findings. A CT scan of her chest (Figure 1) showed peri-bronchial thickening, mucus impaction, and a “tree-in-bud” appearance without bronchiectasis. Bronchoalveolar lavage culture was positive for multidrug-resistant *Pseudomonas*. She was treated with three weeks of ceftazidime and avibactam with aztreonam, in addition to nebulized amikacin to target the resistant *Pseudomonas*.

She was also found to have pancreatic insufficiency (stool elastase was <15 on two occasions) and CFRD, based on a high fasting blood sugar of 16.7 mmol/L (normal value: 4-6 mmol/L). The child was started on a high-calorie diet, pancreatic enzymes, Movicol, multivitamins, and insulin.

Her laboratory workup revealed persistent elevation in alanine aminotransferase (ALT) and aspartate aminotransferase (AST) levels, along with pancytopenia. Her platelet count was 77 × 10⁹/L (normal value: 150-400 × 10⁹/L), WBC was 4.1 × 10⁹/L, and RBC was 4.6 × 10⁹/L. She also had a high prothrombin time of 18.6 (normal value: 12-14), a high international normalized ratio of 1.6 (normal value: 0.8-1.2), and a high partial thromboplastin time of 39 (normal value: 24-36). Abdominal ultrasound showed extensive liver cirrhosis and moderate splenomegaly without convincing features of portal hypertension. A liver biopsy revealed prominent fibrotic changes and histologic features of CFLD. Workups to rule out other causes of chronic liver disease, including viral hepatitis, autoimmune hepatitis, and alpha-1 antitrypsin deficiency, were unremarkable.

**Figure 2:**
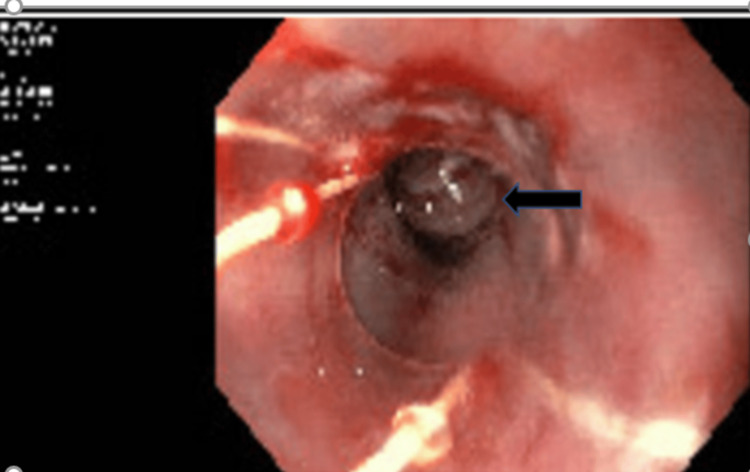
Endoscopy showing esophageal varices after banding

Given the liver cirrhosis and signs of hypersplenism, esophagogastroscopy was performed and identified multiple esophageal varices, which were treated with banding (Figure 2). Following the procedure, she was started on carvedilol, spironolactone, and ursodiol. Follow-up endoscopy showed significant improvement, with only one esophageal varix identified and banded.

**Figure 3:**
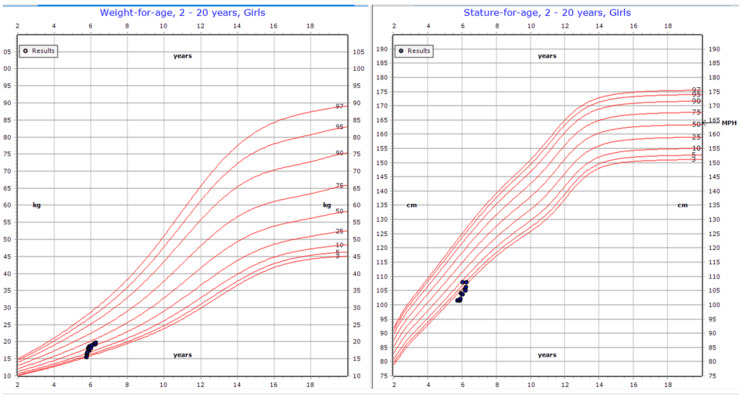
Growth chart showing significant improvement in weight and height over six months

During follow-up appointments over the next six months, she showed significant improvement. Her weight increased from the second to the 34th percentile, with a notable reduction in her abdominal distension (Figure 3). Repeated CBC and liver function tests showed normal ALT and AST levels, with a platelet count of 109 × 10⁹/L.

## Discussion

Our patient has a rare, severe class one genetic mutation associated with advanced liver disease and early-onset CFRD.

CFLD is a known complication of CF, typically emerging within the first 20 years of life and often remaining stable or mildly progressive thereafter [1]. While most children with CF experience some degree of liver steatosis, clinically significant liver disease occurs in fewer than 10% of pediatric CF patients, usually by the age of 10 [2]. It is the third leading cause of death in CF, contributing to only 2-3% of annual fatalities. In the US, approximately 300 CF patients have received liver transplants, with about 75% of these surgeries performed in children [3].

Risk factors for CFLD include male gender, severe mutations, a history of meconium ileus, exocrine pancreatic insufficiency, and CFRD. CFLD predominantly occurs in pancreatic insufficient patients with biallelic loss-of-function mutations in CFTR (class I, II, or III mutations on both alleles). A study in Georgia reported that the 1677delTA mutation was associated with higher rates of growth retardation, liver damage, and CFRD, alongside a lower prevalence of chronic respiratory disease. Additionally, pancreatic insufficiency was notably more severe in their patients [4].

Symptoms of advanced liver disease, such as ascites, splenomegaly, and caput medusa, can be subtle until late in the disease course, by which point cirrhosis may become irreversible and liver transplantation could be required. Nearly all patients with CF-related cirrhosis experience significant malnutrition due to factors such as anorexia, increased catabolism from chronic liver disease, and early satiety due to organ enlargement. Variceal bleeding can occur in both cirrhotic and noncirrhotic patients, often in contexts where hepatic synthetic function is preserved, with varices present in 10-70% of CFLD patients and diagnosed in 25% at CFLD onset. Variceal bleeding is associated with a fivefold increased risk of requiring liver transplantation [5].

As the disease progresses, some patients may develop decompensated cirrhosis, characterized by ascites, liver failure with synthetic dysfunction (such as coagulopathy and hypoalbuminemia), or hepatic encephalopathy [2].

The Cystic Fibrosis Foundation recommends that all individuals with CF undergo annual liver function tests and abdominal examinations. Persistent abnormalities for over six months or the presence of hepatosplenomegaly should prompt a comprehensive abdominal ultrasound with Doppler studies to assess the liver, spleen, and biliary tree and measure portal and hepatic blood flow. A consistent decline in platelet count over time may raise concern, even if thrombocytopenia is not present, and should prompt investigation for portal hypertension. Hepatobiliary ultrasound with Doppler assessments can serve as a noninvasive method for early CFLD detection; however, there is considerable variability in ultrasound imaging, and children with normal results can still have advanced fibrosis. Normal findings, therefore, do not rule out significant liver fibrosis or CFLD [6]. Additionally, Valletta et al. studied echo-Doppler flowmetry for evaluating CF patients at risk of esophageal varices and reported that it may not be reliable in patients with CF and portal hypertension [7]. Our patient was found to have multiple esophageal varices, a complication of portal hypertension, which required endoscopic banding twice despite the absence of evidence of portal hypertension on abdominal ultrasound.

## Conclusions

This case highlights the challenges in diagnosing and managing CFLD, particularly when standard imaging may not detect complications like portal hypertension. Early and thorough screening, including regular liver function tests and abdominal ultrasounds, is crucial to identify and address the progression of CFLD and prevent severe outcomes.
